# Targeting KRAS in NSCLC: Old Failures and New Options for “Non-G12c” Patients

**DOI:** 10.3390/cancers13246332

**Published:** 2021-12-16

**Authors:** Francesca Jacobs, Massimiliano Cani, Umberto Malapelle, Silvia Novello, Valerio Maria Napoli, Paolo Bironzo

**Affiliations:** 1 Department of Oncology, University of Turin, AOU San Luigi Gonzaga, 10043 Turin, Italy; francesca.jacobs@edu.unito.it (F.J.); massimiliano.cani@edu.unito.it (M.C.); silvia.novello@unito.it (S.N.); valeriomaria.napoli@gmail.com (V.M.N.); 2 Department of Public Health, University of Naples Federico II, 80138 Naples, Italy; umbertomalapelle@gmail.com

**Keywords:** KRAS, G12C, oncogene, non-G12C, targeted therapy, NSCLC, drug resistance

## Abstract

**Simple Summary:**

Non-small cell lung cancer (NSCLC) with Kirsten Rat Sarcoma Viral Oncogene Homolog (KRAS) mutation comprises a specific subgroup of patients who are particular in terms of several clinical and molecular aspects. Indeed, there is a clear medical need to find specific and effective treatments for these patients, since KRAS mutation positive NSCLC has demonstrated to be—in some cases—less responsive to standard therapies. For many years, targeting KRAS mutations has been considered an impossible challenge. The scenario is further complicated by the possible role of co-mutations that could influence both tumour microenvironment and drug response. However, some promising preclinical and clinical data are expected to change the treatment landscape of this hard-to-treat disease. Indeed, tumors harbouring G12C mutations could now be effectively targeted with specific inhibitors based on clinical trial results. This review aims to provide a clinical update on potential therapies for advanced NSCLC with KRAS mutations other than the more common G12C, for which good results have already been achieved, particularly focusing on clinical trials, molecules and mechanisms currently under investigation.

**Abstract:**

Kirsten Rat Sarcoma Viral Oncogene Homolog (KRAS) gene mutations are among the most common driver alterations in non-small cell lung cancer (NSCLC). Despite their high frequency, valid treatment options are still lacking, mainly due to an intrinsic complexity of both the protein structure and the downstream pathway. The increasing knowledge about different mutation subtypes and co-mutations has paved the way to several promising therapeutic strategies. Despite the best results so far having been obtained in patients harbouring KRAS exon 2 p.G12C mutation, even the treatment landscape of non-p.G12C KRAS mutation positive patients is predicted to change soon. This review provides a comprehensive and critical overview of ongoing studies into NSCLC patients with KRAS mutations other than p.G12C and discusses future scenarios that will hopefully change the story of this disease.

## 1. Introduction

Approximately 30% of human cancers harbour Kirsten Rat Sarcoma Oncogene Homolog (KRAS) gene mutations, and it is one of the most prevalent alterations. KRAS, together with Neuroblastoma Rat Sarcoma Oncogene Homolog (NRAS) and Harvey Rat Sarcoma Oncogene Homolog (HRAS), belongs to the RAS Oncogenes family, which encodes for small proteins with GTPase activity [1]. KRAS mutations occur in 31% of unresected treatment naïve lung adenocarcinomas [2]. Despite being the most frequent oncogenic alterations, KRAS mutations still represent a high unmet need in solid tumors, including non-small-cell lung cancer (NSCLC). As of today, the “one size fits all” approach still guides the first-line systemic treatment in patients with KRAS mutation (KRAS+) advanced NSCLC. Indeed, patients are treated with immune checkpoint inhibitors (ICIs) alone or in combination with chemotherapy, with a “target agnostic approach”. Recently, structural and biochemical characteristics of mutated KRAS proteins have paved the way for the development of specific inhibitors. Thanks to these efforts, early interesting results have been recently reported in patients harbouring KRAS exon 2 p.G12C mutation with specific tyrosine kinase inhibitors (TKIs) [3]. However, this subgroup encompasses 40–50% of patients with KRAS mutations only, meaning that nearly half of patients with other mutations still lack specific drugs [4]. In this review, we summarise the main attempts, failures, and advances in this field, giving an overview of current clinical studies available today for KRAS “non-p.G12C” patients.

## 2. KRAS Structure and Signalling Pathway

KRAS is involved in multiple cellular pathways [5]. Two isoforms have been identified, deriving from alternative splicing: KRAS4A and KRAS4B, which is the more prevalent [6]. With a molecular weight of 21 kD, RAS proteins are structured in three domains: a hypervariable region, an allosteric lobe, and an effector domain. The latter consists of two switch regions—named I and II—that change their conformation after GTP loading to recruit and activate KRAS effectors [6]. KRAS works by alternating between two states: the active GTP-bound form, and the inactive one linked to GDP [7]. Its activity is regulated by multiple factors, like Guanine Nucleotide Exchange Factors (GEFs) such as Son of Sevenless (SOS). Upon extracellular domain binding, GDP is released, thus facilitating the GTP binding and kinase activation. GTPase activating proteins (GAPs) mediate the conversion of GTP into GDP, bringing KRAS back to the inactive form [8].

KRAS is synthesized in an inactive form within the cytosol. Its tetrapeptide C-terminal CAAX motif is required for a series of post-translational modifications which facilitates its binding to the membrane. The subsequent farnesylation, eased by cytosolic farnesyltransferase (FTase), consists of the addition of a C15 farnesyl isoprenoid to the CAAX motif cysteine, followed by the proteolytic removal, catalysed by Ras-converting enzyme 1 (Rce1) and subsequently by isoprenylcysteine-catalysed carboxymethylation (ICMT) of the terminal farnesylated cysteine [2]. Nevertheless, RAS proteins have another membrane targeting element, consisting of a polybasic amino acid tract (K-Ras4B) or cysteine residues that are reversibly acylated by palmitate fatty acid [2,9].

**Figure 1:**
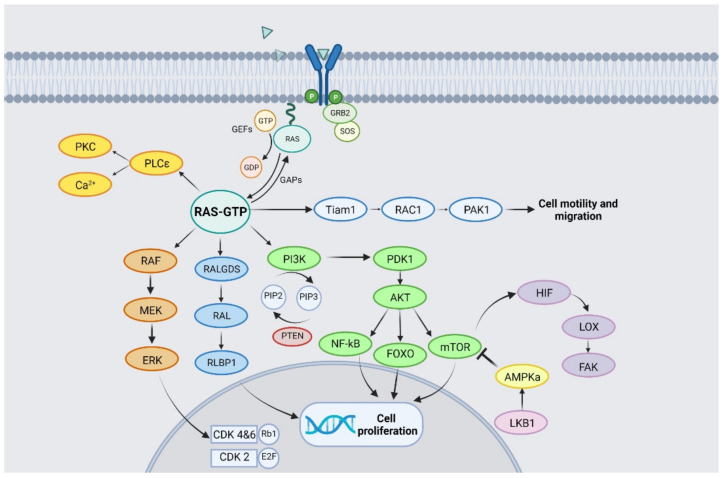
RAS pathway and signalling.

As illustrated in Figure 1, KRAS activates multiple effectors involved in proliferative and mitogenic pathways. KRAS phosphorylates the RAF-kinase, which activates MEK-kinase and is responsible for the activation of the extracellular signal-regulated kinase (ERK). Upon nuclear translocation, ERK triggers several transcription factors responsible for cell proliferation [10].

The KRAS cascade regulates the Phosphatidyl-Inositol 3-Kinase (PI3K/AKT/mTOR) pathway as well [11]. PI3K phosphorylates phosphatydilositol 4,5-biphosphate (PIP2) into phosphatidylinositol 3,4,5-triphosphate (PIP3), which in turn activates PDK1 and its downstream molecule, AKT. The latter has multiple substrates including FOXO, NF-(kb) and the mammalian target of rapamycin (mTOR), all implicated in cell survival and proliferation [10]. Another KRAS effector is RALGDS, a GTP exchange factor, which interacts with the small GTPases RalA and RalB, leading to their activation and therefore contributing to tumorigenesis [12,13]. Although it has theoretical potential, unfortunately no clinical trials are available to date to investigate the targeting of RALGDS.

KRAS also plays a role in cellular metabolism regulation, for instance by influencing glucose metabolism [14]. Alterations in RAS proteins lead to increased aerobic glycolysis and—through the Warburg effect—to anabolic pathways up-regulation with the subsequent production of key elements for cell proliferation. Indeed, AKT pathway activation is increased by methylglyoxal production, while the high glutamine consumption enhanced glutaminolysis enzymes, including NRF2 transcription factor. Hexosamine biosynthesis pathway (HBP) activity is also increased, generating precursors for lipids, protein glycosylation and pentose phosphate cycle. This high glycolytic phenotype is responsible of increased autophagy that enables KRAS-mutated cells to obtain metabolites for cellular sustenance and proliferation. As KRAS-mutated cells need to be continuously supplied with glutamine and glucose to prevent death, therapeutic approaches toward this metabolic vulnerability are under investigation, such as the combination of the KRAS p.G12C inhibitor MRTX849 with mTOR, SHP2 or CDK4/6-directed agents [14]. Finally, changes in lipid metabolism through increased lipogenesis have also been reported [15,16].

## 3. *KRAS* Mutation Subtypes

**Figure 2:**
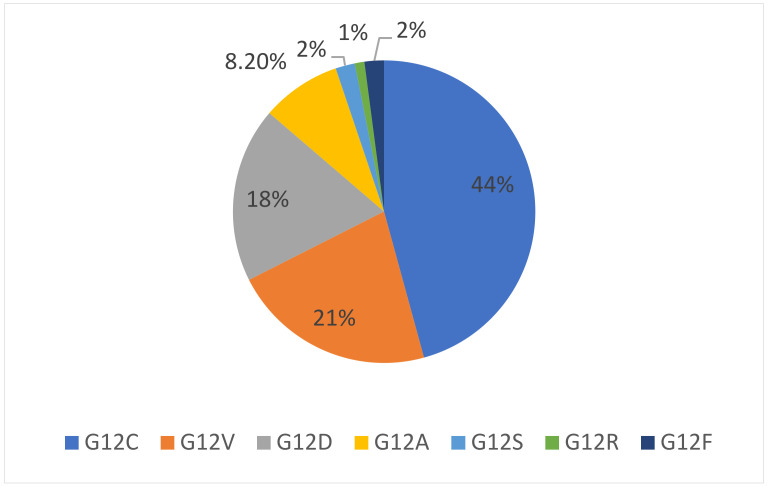
Incidence of KRAS mutation subtypes in NSCLC.

As already mentioned, RAS gene proteins are often mutated in human cancers: NRAS is most frequently mutated in melanoma, KRAS mutations are prevalent in lung, colorectal and pancreatic cancers [7], while HRAS mutations are reported in a minority of cases [17]. KRAS is located on the 12p12.1 chromosome [18] and mutations occur in around 30% of lung adenocarcinomas [19]. Most of them are found at codon 12 (83%) and less commonly at codons 13 (14%), 61, 117 and 146. The most frequent codon 12 alteration is G12C (40%), which consists of a substitution of the native glycine with a cysteine, because of a G>T transversion. G12V mutations represent around 22% of all KRAS mutations, involving the replacement of a glycine by a valine. In the less common G12D mutation (16%), a glycine is replaced by an aspartic acid mutation as a result of a G>A substitution [20]. All KRAS mutation subtypes are summarized in Table 1, while their incidence is represented in Figure 2.

As a consequence of these mutations, the interaction sites of KRAS with GAPs and GEFs are modified, leading to the loss of the GTPase activity, resulting in the activation of the downstream signalling [21].

## 4. *KRAS* Co-Mutations

KRAS mutations are single-driver alterations, so they are usually mutually exclusive with alterations involving Epidermal Growth Factor Receptor (EGFR) and BRAF mutations, or Anaplastic Lymphoma Kinase (ALK) and ROS1 rearrangements, at least at diagnosis [22]. However, co-occurring genomic alterations are reported in up to 50% of KRAS-mutated lung adenocarcinomas. Moreover, they might concur with tumour heterogeneity both in terms of clinical behaviour and response to treatments [4]. Some of them seem to cluster with specific KRAS mutation subtypes, such as those occurring on ERK 1/2 and KRAS G12C [23].

Tumoral protein 53 (TP53) mutations can be found in 39% of KRAS+ NSCLC. Some studies suggest that these mutations could make the cancer more sensitive to immunotherapy by creating a “hot” tumor micro-environment (TME), and by increasing expression of programmed death-1 ligand (PD-L1) and a high tumor mutational burden (TMB) [21,24]. Other frequent co-mutations include Serine/Threonine Kinase 11 (STK11, 20%) and Kelch-like ECH Associated Protein 1/Nuclear Factor Erythroid 2 like 2 (KEAP1/NFE2L2, 13%). KRAS/STK11/KEAP1 co-mutated tumors are characterised by a lower PD-L1 expression and few tumor infiltrating lymphocytes (TILs), leading to a less immune-sensitive TME [23,25]. Moreover, STK11 mutations are associated with a high disease aggressiveness in patients with KRAS exon 2 p.G12D mutation, while its loss of function has been linked to tumor development and progression. Indeed, loss of function of KEAP-1 results in an increased activity of NRF2, a transcription factor involved in cell proliferation and regulation of the pentose phosphate pathway, leading to an alteration in cellular metabolism. NRF2 reprogramming by molecules such as 6-aminonotiamide (6-AN) could control tumour activity, paving the way for specific therapies in this subgroup of patients, usually characterized by poor prognosis [26,27,28].

Indeed, the increased activity of the NRF2 pathway, which is involved in redox balance, leads to a higher glutamine requirement, either due to glutamate consumption (e.g., for reduced glutathione synthesis) or due to increased glutamate-cysteine anti-port system (xCT) activity. This mechanism can be therapeutically exploited using glutaminase inhibitors (such as CB-839), as demonstrated in vitro and in vivo studies in lung cancer cells with an active xCT transporter [29]. The same molecules have been shown to overcome chemotherapy resistance in pancreatic adenocarcinoma cells harboring KRAS mutations. Moreover, glutamine restriction, through GPX4 activity reduction, increased cell sensitivity to chemotherapy in the same model [30].

Alterations involving ATM, and Cyclin Dependent Kinase Inhibitor 2A and 2B (CDKN2A and CDKN2B) have also been found in *KRAS* mutant lung adenocarcinomas [23]. *CDKN2A/B* alterations are frequently associated with the mucinous histology and a low TTF-1 expression, thus being somehow connected to tumor differentiation [25].

In conclusion, recent studies have showed how other genes involved in cellular metabolism, even if not co-mutated, are interconnected to KRAS, like AKT-PI3K and MAPK. This interplay can be exploited in combined therapeutic approaches, as discussed below.

Main co-mutations occurring in KRAS+ NSCLC are summarized in Table 2. 

## 5. Clinical Characteristics and Standard Treatment of *KRAS*-Mutant NSCLC Patients

KRAS+ NSCLC are more frequent among Caucasian patients and are often associated with cigarette smoke, although some mutation subtypes may be found in never-smokers too [35]. The most frequent KRAS mutations among former or current smokers are exon 2 p.G12C and p.G12V, while p.G12D is more frequent among never-smokers [20]. Moreover, *KRAS* mutations (especially p.G12C) are more frequent in women, probably due to an higher susceptibility to cigarette carcinogens [36]. Smoking has also been related to a higher frequency of co-occurring mutations (i.e., TP53 or STK11) [10].

Although KRAS is the most common oncogene driver in NSCLC, no target therapies are approved nowadays in the first line setting of metastatic disease. The current standard of care for KRAS+ advanced NSCLC follows the same treatment algorithms as wildtype tumors. According to the most recent European and American guidelines [37,38], the first-line treatment includes immunotherapy or platinum doublet therapy with immune checkpoint inhibitors (ICIs).

Patients with KRAS+ NSCLC have generally a poorer survival compared to those with wild type or other oncogene-addicted tumors. KRAS subtypes might also influence prognosis and response to pharmacological or surgical therapies. Indeed, KRAS exon 2 p.G12C mutation has been correlated with a worse prognosis after surgical resection, with a higher frequency of recurrence [39].

Despite some contradictory results, most studies do not suggest a predictive role of KRAS mutations in patients treated with chemotherapy (both in the metastatic and adjuvant setting) [40,41,42]. However, recent analyses have shown that KRAS mutations are associated with a shorter overall survival (OS) during chemotherapy treatment [43,44]. Results of the phase III NVALT-22 trial comparing cisplatin plus pemetrexed versus carboplatin plus paclitaxel and bevacizumab in treatment-naïve, advanced, KRAS+ NSCLC patients have been recently presented [45]. Although the study was prematurely closed due to slow accrual, no differences in terms of progression-free survival (PFS, primary endpoint) were observed between arms, suggesting that different platinum-based combinations may be equally active in this patient subgroup.

Even co-mutations may have a prognostic role. STK11 alterations seem to correlate with a shorter overall survival (OS) in KRAS-mutated patients [19], while TP53 mutations predict a worse outcome in patients treated with chemotherapy [46].

The role of KRAS mutations as a predictive factor to ICIs is still unclear. A subgroup analysis of the Checkmate-057 trial, comparing nivolumab with docetaxel in previously treated advanced non-squamous NSCLC, found improved outcomes with immunotherapy in patients harbouring KRAS mutations [47]. Similar results were also found in two recent meta-analyses in the KRAS-mutant subgroup treated with immunotherapy compared to docetaxel in second- and third-line settings [48,49] and in a multi-centre Italian analysis just in terms of 3-months progression-free survival (PFS) [50]. None of these studies have assessed the efficacy of chemo- or immunotherapy considering KRAS mutation subtypes or co-mutations.

Unfortunately, no data are available yet about the predictive role of KRAS/TP53 comutational status when these patients are given a standard combination of chemo- and immunotherapy.

Recently, thanks to the results of CodeBreak 100 and Krystal-1 clinical trials, additional options have been developed for patients harbouring KRAS exon 2 p.G12C mutation who have already received systemic treatments. Two direct inhibitors, sotorasib and adagrasib, have shown good results in phase I and II clinical trials (NCT04625647, NCT04933695, NCT04303780, NCT03785249). On the contrary, therapeutic strategies for “non G12C” KRAS+ NSCLC are still in development.

## 6. Therapeutic Strategies: Recent Clinical Evidence

KRAS has long proven to be a challenging target, mainly due to its structural characteristics, such as its small dimension, its smooth surface and the presence of a single binding site which is occupied by GDP/GTP with a very high affinity [51,52]. The development of effective KRAS inhibitors has long been unsuccessful and continues to be challenging. In recent years, alternative therapeutic approaches have been sought, particularly focusing on mechanisms of downstream inhibition and epigenetic approaches. A systematic breakdown of the most recent data on this subject can be made by subdividing therapies according to the mechanism of action tested. Table 3 depicts an overview of ongoing clinical trials for NSCLC patients with KRAS “non-p.G12C” mutations.

### 6.1. Direct Targeting of Mutant “Non-p.G12C” KRAS

Direct covalent inhibitors have challenged the traditional dogma KRAS+NSCLC as an “undraggable” disease. Thanks to an improved knowledge of KRAS structural and biochemical characteristics, several direct inhibitors were discovered and tested, including SML-8-73-1, ARS-853, ARS-1620 [53,54,55,56,57,58]. Unfortunately, they proved to be effective in KRAS exon 2 p.G12C + tumors only. As of today, no direct inhibitors of “non-p.G12C” mutation-positive tumors are available in clinical trials.

However, some molecules are currently in the pre-clinical stage. Although no published data are yet available, two recent press releases have highlighted the potential of two new small molecules. MRTX1133 is a potent, selective, and reversible inhibitor of KRAS G12D [59], which binds to—and inhibits—mutant KRAS protein both in its active and its inactive state. In in vivo tumor models (including colorectal and pancreatic cancers), this molecule led to tumor regression through a dose-dependent inhibition of the KRAS signalling pathway. A phase I clinical trial is currently being planned. Inhibitors of RAS(ON), which is the active, GTP-bound form of RAS, are also in development [60]. These small molecules drive the formation of complexes that exploit the surfaces of two adjacent proteins to form a new ligand-binding pocket for the inhibitor. Some of these potential new drugs are highly selective for single or a few targets, while others have multi-target activity. For example, RMC-6291 targets KRASG12C/NRASG12C(ON); RMC-6236 targets multiple KRAS variants including KRASG12V(ON) and KRASG12D(ON). These compounds can either be used in combination with other drugs, such as SHP2, mTORC1 or SOS1 inhibitors, to attack multiple targets and potential resistance mechanisms within the RAS pathway simultaneously.

### 6.2. Targeting KRAS Membrane Anchorage

KRAS being a membrane protein, it requires a tight binding to the membrane to be active. As discussed above, membrane binding is dependent on post-translational modification of the CAAX motif by farnesyltransferases. Even if farnesyl-transferases inhibitors (FTIs)—like tipifarnib and lonafarnib—showed some activity in preclinical studies [61,62], clinical results were modest, with response rates lower than 10% [63,64,65]. KRAS prenylation (and thus activation) by geranylgeranyl-transferase-I may explain such results [10]. Newer FTIs are therefore currently under investigation in combination with geranyl-geranyl-transferase inhibitors and showed encouraging activity in pancreatic adenocarcinoma. However, such combinations have not been studied in lung cancer yet [66,67]. Another investigational approach, based on RAS farnesyl cysteine mimetic drugs such as salirasib, despite promising preclinical data [68], did not show any advantage in the clinical setting [69].

Bisphosphonates and inhibitors of enzymes directly involved in prenylation, such as the RAS-converting CAAX endopeptidase 1 (Rce1) and the isoprenylcisteine carboxyl methyltransferase (ICMT), have been studied in Ras-driven tumors. Despite preclinical evidence, none of these approaches have been proven to be effective [70,71].

### 6.3. Targeting KRAS Downstream Pathways

The KRAS pathway is particularly complex, involving many other signalling pathways, such as the RAF-MEK-ERK and the PI3K/AKT/mTOR ones. Sorafenib, a multi-kinase inhibitor with activity against many protein kinases, including Vascular Endothelial Growth Factor Receptor (VEGFR), Platelet-derive Growth Factor Receptor (PDGFR) and RAF kinase, showed preclinical activity [72]. A phase II study of sorafenib [73] enrolled pre-treated patients with *KRAS*+, stage IIIB or IV NSCLC. The disease control rate at 6 weeks (primary endpoint of the study) was 52.6%, with a median duration of treatment of 9 weeks. The median PFS was 2.3 months, and the median OS was 5.3 months. These data led to the phase III, randomized MISSION trial [74] of sorafenib versus placebo as a third- or fourth-line therapy of NSCLC patients. Despite patients being enrolled regardless of mutational status, *EGFR* and KRAS mutations were analysed on tissue or blood samples. A modest PFS benefit was observed in the experimental arm both in the overall population (2.8 vs. 1.4 months, *p* < 0.0001) and in the KRAS+ subgroup (2.6 vs. 1.7 months, *p* = 0.007). However, there were no differences in mOS (primary efficacy endpoint were obtained with sorafenib neither in the overall population (8.2 vs. 8.3 months, *p* = 0.47) nor in the KRAS+ subgroup (6.4 vs. 5.1 months, *p* = 0.279).

Following encouraging phase II data [75], the MEK inhibitor selumetinib was tested in association with docetaxel in the large phase III SELECT-1 trial. This multinational, randomized, clinical trial enrolled patients with advanced KRAS+ NSCLC who had progressed after first-line therapy. The study was negative, as it failed to show any differences in terms of PFS between arms (mPFS 3.9 versus 2.8 months, *p* = 0.44) [76]. Selumetinib was also evaluated in combination with the EGFR-TKI erlotinib in a phase II study of advanced KRAS+ NSCLC. The combination did not improve PFS and led to several toxicities [77].

A phase II study comparing the MEK inhibitor trametinib with docetaxel involving pre-treated, advanced, KRAS+ NSCLC was also negative (mPFS 12 vs. 11 weeks; *p* = 0.5197) [78]. Interestingly, another phase II study that explored the combination of trametinib and docetaxel in 54 KRAS+ advanced, pre-treated NSCLC patients showed a response rate of 33% and a mOS of 11.1 months. Even if the difference in response rate (RR) between mutation subtypes was not statistically significant, there was a trend for worse PFS and OS in the G12C subgroup [79].

A phase Ib/II trial (NCT02079740) is currently investigating the potential of a combination of trametinib and the BCL-XL inhibitor navitoclax based on preclinical data [80]. Early results have shown that, at the recommended phase 2 dose (RP2D), RR was 15.4% with a disease control rate of 46.2% [81]. Expansion cohorts are currently enrolling patients with solid tumours, including NSCLC, and further data are awaited (Table 3).

One possible explanation for the failure of MEK inhibitors as single agents could rely on alternative downstream pathways activation. To tackle such resistance mechanisms, some early phase studies combined PI3K or mTOR inhibitors, with encouraging results. However, the toxicity profile precluded further investigation of such combinations [82,83,84]. Currently, clinical trials are underway to investigate the potential therapeutic role of MEK inhibitors combined with cyclin inhibitors, FAK inhibitors, ICIs directed against programmed death protein 1 (PD-1), and other small molecules (Table 3).

Recently, interesting data came from the investigation of VS-6766 (previously known as CH5126766 and RO5126766), a small molecule that inhibits both MEK and RAF kinases [85]. One of the critical points about MEK inhibition is that it paradoxically induces MEK phosphorylation by relieving ERK-dependent feedback inhibition of RAF [86,87]. VS-6766 can disrupt the formation of RAF–MEK complexes, which would otherwise reactivate MEK [85]. A phase 1b dose-escalation, basket expansion study of VS-6766 as a single agent [88] showed promising anti-tumor activity in *KRAS* non-p.G12C mutant patients with solid tumors (including NSCLC) and multiple myeloma. Three out of the 10 NSCLC patients had a partial response, and all these responses were maintained for at least 6 months. Of note, 2 of these patients had *KRAS* exon 2 p.G12V mutation. Notably, *KRAS* exon 2 p.G12V mainly exploits the RAF/MEK cascade, in contrast to other variants—such as p.G12D—whose signal is preferentially mediated by PI3K/AKT [89]. More specifically, *KRAS* exon 2 p.G12V models are especially dependent on CRAF, suggesting the role of VS-6766 in this molecularly defined disease [86,87,88,89]. Anyway, a stronger inhibitor of tumor growth requires more complex strategies. The combination of VS-6766 and the FAK inhibitor defactinib was shown to overcome FAK phosphorylation induced by MEK inhibition in in vivo models [90,91]. An open label phase I dose escalation with an expansion study (FRAME trial—NCT03875820) is recruiting patients with advanced, *KRAS*+, solid tumors to evaluate this combination. Data about the NSCLC patient subgroup were recently presented at the 2021 American Association for Cancer Research Annual Meeting [92]. The median prior lines of treatment were 3, and all patients had previously received ICIs. Seventeen out of 19 patients were evaluable for a response. Of them, 2 (12%) had a partial response and 10 (59%) had a stable disease. Notably, 11 patients (65%) achieved some degree of tumor size reduction and 5 (29%) remained on treatment for at least 6 months. Interestingly, 2/2 (100%) of the *KRAS* NSCLC patients achieved a partial response. This combination has so far showed a favourable tolerability profile with the intermittent dosing regimen investigated in the FRAME trial. The incidence of adverse events of Grade ≥ 3 was 5%. Based on these promising results, a phase Ib/II study evaluating VS-6766 with or without defactinib for the treatment of *KRAS* (especially G12V subtype) +, advanced, NSCLC (NCT04620330) was initiated in December 2020 and is currently recruiting patients.

Because of the PI3K/AKT/mTOR pathway involvement in KRAS downstream signalling, and given the synergy of this association in cancer cell lines harbouring various subtypes of KRAS mutations, VS-6766 is also being evaluated in combination with the mTOR complex1 (mTORC1) inhibitor everolimus in a phase I study (NCT02407509).

Unfortunately, results coming from clinical studies about PI3K/AKT/mTOR inhibitors as monotherapy have so far been disappointing. Ridaforolimus, a selective mTOR inhibitor, showed an objective response rate (ORR) of only 1% with high toxicity rate [93]. The phase II BASALT-1 trial explored a potential role of buparlisib (BKM120, a pan-PI3K inhibitor) in advanced, pre-treated, NSCLC patients with an activated PI3K pathway. The study was closed early in stage 1 for futility. However, a longer PFS was observed in a subgroup of 12 patients also harbouring a KRAS mutation [94].

Another potential therapeutic approach relies on Src Homology region 2 (SH2)-containing protein tyrosine phosphatase 2 (SHP2) inhibition. RMC-4630 and TNO155 are two SHP2 inhibitors in clinical development alone or in combination with other drugs (NCT04000529; NCT04916236; NCT03114319; NCT03989115) (Table 3). Preliminary results of the phase I trial of RMC 4630 showed a disease control rate (DCR) of 67% among the 19 *KRAS*+ NSCLC patients. A higher response rate was observed in 7 patients with *KRAS* G12C mutation (71%) [95].

In conclusion, inducing KRAS degradation could be an alternative way of controlling tumor growth. With this intent, Bery et al. established an in vitro and in vivo murine model, in order to evaluate KRAS degradation activity of two types of macromolecules [96]. The first one, ankyrin repeat proteins (DARPins) K13 and K19 engineered with E3-ligase, can interfere with KRAS, causing its degradation, while the second one, an engineered pan-RAS binding intracellular single domain antibody (iDAbs), is active against all RAS forms. This study showed that both molecules cause RAS degradation, but K19 DARPins inhibit cell proliferation without interfering with wild-type KRAS cells. Moreover, Shin et al. evaluated a novel RAS inhibitor, KY7749, that is able to degradate KRAS G12V in a beta-catenin-independent manner in in vitro colorectal cancer models [97].

### 6.4. Targeting Co-Dependent Vulnerabilities or Synthetic Lethal Partners

Co-dependent vulnerabilities and synthetic lethal partners are necessary for *KRAS* oncogenesis and tumour growth [98]. Bortezomib, a proteasome inhibitor, was investigated in a phase II clinical trial including only patients with *KRAS* exon 2 p.G12D + advanced NSCLC. The disease control rate (DCR) was 40%, while the mPFS and OS were 1 month and 13 months, respectively [99].

Cyclin-dependent kinase (CDK) inhibition has also been considered as a potential therapeutic approach. Abemaciclib, a CDK4/6 inhibitor, showed promising data in the multicentre, randomized, phase III, JUNIPER trial. This trial randomized advanced, pre-treated, NSCLC patients harbouring *KRAS* codon 12 or 13 mutations to receive abemaciclib with best supportive care (BSC) or erlotinib with BSC. The study primary end-point was not met as mOS was 7.4 months with abemaciclib and 7.8 months with erlotinib (*p* = 0.77). However, both mPFS and ORR were higher with abemaciclib. [100,101]. Other CDK inhibitors are currently under investigation in combination with other agents in KRAS+ NSCLC (NCT03170206, NCT02022982NCT02974725) (Table 3).

Inactivating LKB1/STK11 mutations generally cause poor response to anti-PD-1 monoclonal antibodies, even if they correlate with an elevated tumor mutational burden (TMB) and an increased number of neoantigens [102]. LKB1 is frequently co-mutated with KRAS and it leads to an objective response rate to immunotherapy of less than 10%. LKB1 deficiency suppresses antigen processing and presentation because it compromises immunoproteasome activity, thus enhancing the autophagic flux, mediated by the autophagy-lysosomal pathway [103]. The combination of a Unc-51 Like Autophagy Activating Kinase 1 (ULK1) inhibitor, MRT68921, with an anti-PD-1 monoclonal antibody can increase antigen presentation and therefore restore anti-tumor immunity, by compensating for LKB1 loss, leading to KRAS/LKB1 co-mutant tumor regression [102]. This seems to be due to the immunoproteasome activity enhancement obtained by ULK1 inhibition. Based on these preliminary results, this combination deserves clinical evaluation in patients whose tumors harbour both KRAS and LKB1 mutations.

### 6.5. Targeting KRAS Activity Regulators

Interaction with guanine nucleotide exchange factors (GEFs) is necessary for RAS activation. SOS, which catalyses the release of GDP and the binding of GTP, is one of such factors. BI1701963 is a small molecule that binds and inhibits SOS1, leading to a KRAS blockade regardless of the mutation subtype [104,105,106]. Early data about BI1701963, presented at the 2019 AACR-NCI-EORTC International Conference on Molecular Targets and Cancer Therapeutics, led to an ongoing phase I clinical trial that is exploring this drug both as monotherapy and in combination with trametinib in patients with advanced, *KRAS*+, solid tumors (NCT04111458).

### 6.6. Targeting Immune System

There is a strong rationale that led to the evaluation of ICIs in KRAS+ NSCLC. Indeed, RAS pathway has several immunomodulating effects, including regulation of CD8+ lymphocytes infiltrate, myeloid derived stem cells (MDSCs) density and PD-L1 expression [107]. Although data regarding the role of immunotherapy in this subgroup of patients are still controversial, as previously discussed [48,50], several studies are currently evaluating combinations of ICIs with small molecules. A phase Ib/II trial evaluating the anti PD-L1 monoclonal antibody avelumab combined with the MEK1/2 inhibitor binimetinib, with or without the PARP inhibitor talazoparib (NCT03637491), including patients with advanced, *KRAS* + NSCLC, showed limited anti-tumor activity with a high toxicity. The phase II BATTLE-2 trial is evaluating an association between pembrolizumab and trametinib in advanced, pre-treated, NSCLC patients, unselected for *KRAS* mutational status (NCT03225664).

The combination of FAK inhibitors with ICIs would also be intriguing, as FAK is also involved in the immune system regulation. Indeed, its inhibition reduces stromal density, enhancing the cytotoxic T cell entry and reducing immunosuppressive Tregs, M2 macrophages and MDSCs [108]. As of today, no clinical trials are evaluating such combinations. The high immunogenicity of KRAS suggests a possible role of adaptive immunity approaches too. Early interesting data suggest the in vitro efficacy of p.G12V-reactive T cells against this subtype of KRAS mutation [21,109].

Cancer vaccines may be another approach for pan-RAS targeting. Following the pre-clinical evidence that lymph node-targeted vaccines could stimulate a robust immune response against the mutant KRAS protein in mice [110], a phase I/II trial (NCT04853017) evaluating the safety and preliminary efficacy of ELI-002 has been recently activated. This drug is an investigational, subcutaneous, amphiphile (AMP) KRAS therapeutic vaccine made of seven AMP-modified mutant KRAS peptide antigens and ELI-004, an AMP-modified immune-stimulatory oligonucleotide adjuvant. ELI-002 can therefore target all seven position 12 and 13 KRAS mutations. The property AMP platform has been specifically designed to deliver investigational immunotherapeutics directly to the lymph nodes. ELI-002 latches on to the protein albumin, found in the bloodstream, as it travels to lymphatic tissue. Different to other vaccines, this enables a precise targeting and delivery of immunogens and cell-therapy activators directly to the “control unit” of the immune system, to significantly amplify its activity. Once the AMP mKRAS peptides and AMP CpG have reached the lymph node, amphiphile immunotherapies are taken up by antigen-presenting cells (APCs) and can generate RAS-specific killer T cells directed against tumor cells. In this trial, ELI-002 will be administered to patients with KRAS+ solid tumors, including NSCLC, and minimal residual disease (MRD) after surgical resection. Circulating tumour DNA (ctDNA) will be assessed to monitor the presence of MRD and the vaccine activity. Although this vaccine may be extremely precise and active, based on preclinical data, one major concern is the duration of the immunity. Clinical studies will be crucial to assess the benefits and limitations of this novel promising treatment approach.

### 6.7. Epigenetic Approaches

RAS/MEK pathway activation can increase telomerase activity and telomeres length [111]. Clinical data with epigenetic drugs are available for unselected NSCLC cohorts only, possibly diluting effects based on *KRAS* mutational status, if any.

A phase II study explored a potential role for the telomerase inhibitor imetelstat as maintenance therapy. The study enrolled patients with non-progressive, advanced NSCLC after first-line, platinum-based chemotherapy, with or without bevacizumab and randomized them 2:1 to imetelstat or observation. No differences in terms of PFS nor OS were observed in the overall population, while a trend towards improved survival was seen in patients with a short telomere length [112].

Chromatin-modifying agents, such as the hystone deacetylase inhibitor (HDACi) panobinostat, have also been investigated based on preclinical study results [113]. Vorinostat, another HDACi, was evaluated in a phase II trial [114] in association with carboplatin and paclitaxel. The experimental combination showed a significantly higher RR (34% vs. 12%; *p* = 0.02), while no statistically significant differences in terms of PFS and OS were observed.

## 7. Discussion

Despite recent progress made on targeting KRAS exon 2 p.G12C mutations in advanced NSCLC, effective treatments for other KRAS subtypes are still lacking. While the road towards direct inhibitors in “non-p.G12C” KRAS + NSCLC seems still long and winding, combination therapies against downstream pathways hold promise. On the other hand, even novel immunotherapy approaches are entering the clinical research arena, enlarging the investigational agent portfolio. As each KRAS mutation subgroup behaves differently, only a thorough study of both biological and clinical characteristics of different KRAS+ populations would speed up research leading to novel and active targeted approaches, even in such hard-to-treat diseases.

In this complex scenario, there is an urgent need for collaborative efforts between different institutions to develop knowledge-based databases. These may help the different healthcare figures involved in the adequate management of NSCLC patients, avoiding any delay or misunderstanding. Regarding RAS mutations, in a recent experience, Malapelle et al. collected real world practice data, obtained by 12 referral Italian institutions related to lung and colon cancer biomarker testing [1]. In particular, the total volume of tested samples, mutations identified, and assays adopted for molecular analysis were retrieved by each center. As a matter of fact, all these data were compared with those reported in the COSMIC database in order to develop a user-friendly knowledge-based database (www.rasatlas.com (accessed on 9 December 2021)) that was useful to permit the best treatment option and management for *RAS* gene-mutated patients.

## 8. Conclusions

In recent years, many approaches have been attempted to find effective treatment options for NSCLC with KRAS mutation, a disease that has remained without effective options for years. After many disappointments and some promising results that have shed light on potential therapeutic targets, the greatest certainty is that only a better understanding of these extremely complex mechanisms, together with an increased knowledge of the structural features of the diverse mutation subtypes and co-mutation profiles, will hopefully guide us to the overcoming of resistance and thus to a true therapeutic efficacy.

## Figures and Tables

**Table 1:** KRAS mutation subtypes and their incidence in lung adenocarcinoma.

Mutation Subtype	Codon 12 Transition or Transvertion	Aminoacid Substitution	Mutation Frequency in Lung Adenocarcinoma
G12C	G>T transversion resulting in GGT to TGT	Glycine to cysteine	44%
G12V	G>T transversion resulting in GGT to GTT	Glycine to valine	18–21%
G12D	G>A transition resulting in GGT to GAT	Glycine to aspartic acid	11–18%
G12A	G>C transvertion resulting in GGT to GCT	Glycine to alanine	8.2%
G12S	G>A transition resulting in GGT to AGT	Glycine to serine	2%
G12R	G>C transversion resulting in GGT to CGT	Glycine to arginine	1%
G12F	GG>TT transversion resulting in GGT to TTT	Glycine to phenylalanine	2%

**Table 2:** Co-mutations in KRAS mutant NSCLC.

Co-Mutated Genes	Gene- Location	Frequency	Main Features	Ref
TP53	17p13.1	39%	Shorter latency and greater metastatic tendency; high expression of PD-L1 and TILs in TME; good response to immunotherapy.	[23,31]
STK11	19p13.3	20%	Greater tumour growth rate, increased tendency to turn into squamous histology; low expression of PD-L1 levels and reduced TILs in TME; reduced response to chemo-immunotherapy.	[21,23,25,32]
KEAP1	19p13.2	13%	Increased tumour progression rate; low response to platinum-based chemotherapy and immunotherapy.	[23,25,33]
ATM	11q22.3	11.9%	Incomplete ATM loss is associated to carcinogenesis in case of p53 deficiency.	[23]
CDKN2A and CDKN2B	9p21.39p21.3	20% 12%	Associated to mucinous histology with a lower TTF-1 expression.	[23,25,34]

TP53 = Tumor Protein P53; PD-L1 = Programmed Death-Ligand 1; TILs = Tumor-Infiltrating Lymphocites; TME = Tumor Micro Environment; STK11 = Serine/threonine kinase 11; KEAP1 = Kelch-like ECH-associated protein 1; ATM = Ataxia-Telangiectasia Mutated; CDKN2A = Cyclin Dependent Kinase Inhibitor 2°; CDKN2B = Cyclin Dependent Kinase Inhibitor 2B; TTF-1 = Thyroid Transcription Factor 1.

**Table 3:** Which options for “non-G12C” KRAS patients? Main ongoing clinical trials (clinicaltrials.gov (accessed on 20 July 2021)).

Clinical Trial	Drug(s) Name	Target	Phase	Population and Tumor Characteristics	Estimated or Actual Enrollment	Status
NCT03875820 (FRAME)	VS-6766 plus defactinib	MEK/RAF, FAK,	1	Solid advanced tumours, including KRAS mutant NSCLC	80	Recruiting
NCT04620330 (RAMP-202)	VS-6766 plus defactinib	MEK/RAF, FAK	1b/2	Advanced, pre-treated, G12V or other KRAS mutant NSCLC	100	Recruiting
NCT02407509	VS-6766 plus everolimus	MEK/RAF, mTOR	1	Solid advanced tumours, including KRAS mutant NSCLC, and multiple myeloma	94	Recruiting
NCT03681483	VS-6766	MEK/RAF	1	Advanced KRAS mutant lung cancer	15	Active, not recruiting
NCT03704688	Ponatinib + trametinib	Bcr-Abl, MEK/MAPK/ERK	1b/2	Previously treated KRAS mutant advanced NSCLC	12	Active, not recruiting
NCT02964689	Binimetinib + pemetrexed and cisplatin, followed by maintenance with binimetinib + pemetrexed	MEK	1	Advanced KRAS mutant NSCLC	18	Active, not recruiting
NCT03990077	HL-085 + docetaxel	MEK	1	Advanced pre-treated KRAS mutant NSCLC	27	Not yet recruiting
NCT01912625	Trametinib + chemoradiation	MEK	1	Stage III NSCLC that cannot be removed by surgery	16	Active, not recruiting
NCT02079740	Trametinib + navitoclax	MEK, BCL-XL	1b/2	Solid advanced tumours with KRAS mutation, including NSCLC	130	Recruiting
NCT04735068	Binimetinib + hydroxychloroquine	MAPK, lysosome	2	Advanced KRAS mutant NSCLC	29	Not yet recruiting
NCT04566393	Expanded Access to ulixertinib (BVD-523)	MAPK pathway	-	Advanced solid tumours (including NSCLC) with MAPK pathway alterations, including KRAS mutations	-	Expanded Access Avalaible
NCT02022982	Palbociclib + PD-0325901	CDK4/6, MEK	1b/2	Solid cancers with KRAS mutations, including NSCLC	139	Active, not recruiting
NCT03170206	Palbociclib (PD-0332991) and binimetinib (MEK162)	CDK4/6, MEK	1b/2	Advanced KRAS mutant NSCLC	72	Recruiting
NCT02974725	LXH254 + LTT462 or trametinib or ribociclib	RAF, ERK/MEK/CDK4/6	1b	Advanced solid tumours, including KRAS mutant Non-Small Cell Lung Cancer	331	Recruiting
NCT03299088	Pembrolizumab + trametinib	PD-1, MEK	1	Advanced KRAS mutant NSCLC	15	Active, not recruiting
NCT03225664 (BATTLE-2)	Pembrolizumab + trametinib	PD-1, MEK	1b/2	Advanced, previously treated NSCLC	37	Active, not recruiting
NCT01859026	Binimetinib (MEK162) + erlotinib	MEK, EGFR	1/1b	Advanced NSCLC harbouring KRAS or EGFR mutation	43	Active, not recruiting
NCT03520842	Regorafenib + methotrexate	Multiple kinases	2	Recurrent or metastatic KRAS mutated NSCLC	18	Recruiting
NCT04000529	TNO155 + spartalizumab or ribociclib	SHP2, PD-1, CDK4/6	1	Solid advanced tumours, including KRAS mutant NSCLC	126	Recruiting
NCT04916236 (SHERPA)	RMC-4630 + LY3214996	SHP2, ERK	1	KRAS mutant cancers, including NSCLC	55	Not yet recruiting
NCT03114319	TNO155	SHP2	1	Advanced solid tumours, including KRAS G12-mutant NSCLC	255	Recruiting
NCT03989115	RMC-4630 + cobimetinib	SHP2, MEK	1b/2	Advanced solid tumours, including KRAS G12-mutant NSCLC	168	Recruiting
NCT03808558	TVB-2640	FAS/FASN	2	Advanced KRAS mutant NSCLC	12	Recruiting
NCT03965845	Telaglenastat (CB-839) + palbociclib	Glutaminase, CDK4/6	1b/2	Solid advanced tumours, including pre-treated, KRAS mutant NSCLC	85	Recruiting
NCT04263090	Rigosertib + nivolumab	PI3K/PLK, PD-1	1	Advanced, pre-treated, KRAS mutant NSCLC	30	Recruiting
NCT03693326	PDR001	PD-1	2	Non-small Cell Lung Cancer harbouring mutations including KRAS	70	Recruiting
NCT04470674	Carboplatin-pemetrexed +/− durvalumab	PD-L1	2	Advanced, naïve, KRAS mutant and PD-L1 high (≥50%) NSCLC	50	Recruiting
NCT03777124	SHR-1210 + apatinib	PD-1, VEGFR2	2	KRAS mutant stage IV non-squamous NSCLC	230	Not yet recruiting
NCT04853017 (AMPLIFY-201)	ELI-002 (lymph node-targeted therapeutic vaccine)	-	1/2	KRAS/NRAS mutant (G12D or G12R) solid tumours, including NSCLC	159	Recruiting
NCT03095612	Selinexor (KPT-330)	XPO1	1b/2	Pre-treated advanced KRAS mutant lung cancer	59	Recruiting
NCT03819387	NBF-006 (siRNA-based lipid nanoparticle)	-	1	Solid advanced tumours including KRAS-Mutant NSCLC	44	Recruiting
NCT03948763	mRNA-5671/V941 +/− pembrolizumab	-	1	Solid advanced tumours, including KRAS mutant NSCLC	100	Recruiting
